# Redox Status and Aging Link in Neurodegenerative Diseases

**DOI:** 10.1155/2014/270291

**Published:** 2014-03-20

**Authors:** Verónica Pérez de la Cruz, Sathyasaikumar V. Korrapati, José Pedraza-Chaverrí

**Affiliations:** ^1^Departamento de Neuroquímica, Instituto Nacional de Neurología y Neurocirugía Manuel Velasco Suárez, S.S.A., 14269 México, DF, Mexico; ^2^Maryland Psychiatric Research Center, University of Maryland School of Medicine, Baltimore, Maryland, MD 21228, USA; ^3^Departamento de Biología, Facultad de Química, Universidad Nacional Autónoma de México, 04510 México, DF, Mexico

Longevity is a complex and multifactorial process in which the balance in redox state has an important role. The production of reactive oxygen/reactive nitrogen species (ROS/RNS) at low level is involved in physiological process as signaling molecules in various cellular and developmental processes. However, the increase in the production of these species and/or the decrease in the antioxidant capacity can lead to perturbation of the redox balance, causing oxidative/nitrosative stress which ultimately leads to cell death. The aging and neurodegenerative diseases are closely associated with the unbalance in the redox environment. This special issue contributes to the understanding of the impact of impairment in the balance of antioxidant defense and ROS/RNS generation as well as the mechanisms that are involved, which will lead to a better comprehension of several processes related to aging and neurodegeneration biology.

Three review articles are focused on Alzheimer's disease. D. Luque-Contreras et al. reviewed the role of extracellular *β*-amyloid, tau, and apoE, three main proteins associated with the development of Alzheimer's disease, in the generation of oxidative stress as well as the mitochondrial alterations induced by extracellular *β*-amyloid and its relationship with vascular damage. Moreover, P. Carrillo-Mora et al. mentioned that although there is a lot of information of the possible mechanisms that may be involved in the role of *β*-amyloid, its participation in the pathophysiology of Alzheimer's disease is not fully understood. The authors described both toxic (excitotoxicity, mitochondrial alterations, synaptic dysfunction, and altered calcium homeostasis) and some neuroprotective mechanisms of *β*-amyloid* in vitro* and* in vivo*, which may contribute to the understanding of the processes in which this protein is present and lead to the design of future therapeutic interventions. Additionally, M. A. Meraz-Ríos and coworkers provide a review of the mutations present in APP, PS1, and PS2 observed in patients with familial Alzheimer's disease and its association with oxidative stress.

M. Luca and coworkers describe the importance of nitrooxidative stress as an important factor in the aging and neurodegeneration and as a link of all these factors with major depression and also the authors correlated these factors with cell functioning, gene expression, and proteins folding.

The research article by S. González-Reyes and coworkers shows the protective effect of curcumin against hemin-induced damage in primary cultures of cerebellar granule neurons. Specifically, the mechanisms by which curcumin can show its benefic properties are through Nrf2 modulation and the induction of antioxidant response. In the same context, the original article of M. Otero-Losada and coworkers demonstrates that an antioxidant supplementation with alpha-tocopherol, *β*-carotene, and vitamin C improved the biochemical profile associated with oxidative metabolism in elderly cardiovascular patients. These original articles represent excellent evidence in which the modulation of antioxidants response can be seen as a fertile line to explore both experimental and clinical studies in which the oxidative stress is an important factor.

Also the review of N. Cárdenas-Rodriguez and coworkers describes how the antiepileptic drugs as valproic acid, oxcarbazepine, and topiramate modulate antioxidant status by modifying the activity of antioxidant enzymes both in human and animals models and these effects can be also independent of their principal mechanism of action.

D. Silva-Adaya and coworkers introduced for us an interesting review that focuses on thioredoxin system (thioredoxin, thioredoxin reductase, and NADPH) expression into the central nervous system. The authors also described the conditions that modulate the thioredoxin system in both animal models and the postmortem brains of human patients associated with the most common neurodegenerative disorders, in which this system could play an important role.

On the other hand, J. R. Ocampo and coworkers discuss the redox properties of kynurenine pathway metabolites which have been involved in many brain diseases and aging. They also describe the effect of these kynurenines under different conditions since the environment can modify their activity. Additionally, the authors reviewed the changes in the levels of these metabolites during the age and some brain disorders; the review also takes the evidence in the literature and explains the possible impact that these kynurenines levels alteration can have on the NAD^+^ production.

In the review article of S. Montes and coworkers was summarized updated information regarding copper and copper proteins in Parkinson's disease. Some evidences have revealed that transition metals play important roles in the setup and development of neurodegenerative diseases; copper is a special case, since its physiological actions influence other metals, such as iron. The specific reports regarding Parkinson's disease (and experimental models) and copper-related proteins, ceruloplasmin, superoxide dismutase, and metallothionein, are reviewed in detail. The participation of copper transporters and the relationship of this transition metal and Parkinson's disease-related proteins such as alpha synuclein are also reviewed. Evidence suggests that antioxidant function of copper proteins could be an interesting experimental strategy to test neuronal death in Parkinson's disease.

The original article by A. Diaz-Ruiz and coworkers shows the metallothionein-II capability to inhibit lipid peroxidation and to improve functional recovery after transient brain ischemia and reperfusion in an experimental model in rats using biochemical, functional, and histological evaluations. Their results suggest that MT-II may be a neuroprotective treatment to prevent tissue damage after cerebral ischemia.

Thyroid hormones are related to oxidative stress not only by their stimulation of metabolism but also by their effects on antioxidant mechanisms. I. Villanueva and coworkers analyzed the participation of thyroid hormones on ROS production and oxidative stress and the way the changes in thyroid status in aging are involved in neurodegenerative diseases.

Together all the papers of this special issue provide a better understanding of the mechanisms involved during the process of aging and brain diseases, which could be potential therapeutic targets in the future.

